# Legacy effects of anaerobic soil disinfestation on soil bacterial community composition and production of pathogen-suppressing volatiles

**DOI:** 10.3389/fmicb.2015.00701

**Published:** 2015-07-10

**Authors:** Maaike van Agtmaal, Gera J. van Os, W.H. Gera Hol, Maria P.J. Hundscheid, Willemien T. Runia, Cornelis A. Hordijk, Wietse de Boer

**Affiliations:** ^1^Department of Microbial Ecology, Netherlands Institute of Ecology, NIOO-KNAWWageningen, Netherlands; ^2^Applied Plant Research, Flowerbulbs, Nursery Stock and Fruit, Wageningen University and Research CentreLisse, Netherlands; ^3^Department of Terrestrial Ecology, Netherlands Institute of Ecology, NIOO-KNAWWageningen, Netherlands; ^4^Applied Plant Research, Subdivision Arable Farming, Multifunctional Agriculture and Field Production of Vegetables, Wageningen University and Research CentreLelystad, Netherlands; ^5^Department of Soil Quality, Wageningen University and Research CentreWageningen, Netherlands

**Keywords:** volatile organic compounds (VOCs), Soil-borne plant pathogens, General disease suppression, Pythium intermedium, Fungistasis, Oomycetes

## Abstract

There is increasing evidence that microbial volatiles (VOCs) play an important role in natural suppression of soil-borne diseases, but little is known on the factors that influence production of suppressing VOCs. In the current study we examined whether a stress-induced change in soil microbial community composition would affect the production by soils of VOCs suppressing the plant-pathogenic oomycete *Pythium*. Using pyrosequencing of 16S ribosomal gene fragments we compared the composition of bacterial communities in sandy soils that had been exposed to anaerobic disinfestation (AD), a treatment used to kill harmful soil organisms, with the composition in untreated soils. Three months after the AD treatment had been finished, there was still a clear legacy effect of the former anaerobic stress on bacterial community composition with a strong increase in relative abundance of the phylum *Bacteroidetes* and a significant decrease of the phyla *Acidobacteria, Planctomycetes, Nitrospirae, Chloroflexi*, and *Chlorobi*. This change in bacterial community composition coincided with loss of production of *Pythium* suppressing soil volatiles (VOCs) and of suppression of *Pythium* impacts on Hyacinth root development. One year later, the composition of the bacterial community in the AD soils was reflecting that of the untreated soils. In addition, both production of *Pythium*-suppressing VOCs and suppression of *Pythium* in Hyacinth bioassays had returned to the levels of the untreated soil. GC/MS analysis identified several VOCs, among which compounds known to be antifungal, that were produced in the untreated soils but not in the AD soils. These compounds were again produced 15 months after the AD treatment. Our data indicate that soils exposed to a drastic stress can temporarily lose pathogen suppressive characteristics and that both loss and return of these suppressive characteristics coincides with shifts in the soil bacterial community composition. Our data are supporting the suggested importance of microbial VOCs in the natural buffer of soils against diseases caused by soil-borne pathogens.

## Introduction

In the light of sustainable agriculture and the call for reduction of pesticide use, insights in the mechanisms of natural suppression of soil-borne pathogens are essential. Therefore, understanding the interactions of plant pathogens with other members of soil microbial communities is needed to develop strategies for effective and consistent control (Chaparro et al., 2012). In general, depletion of carbon sources by indigenous microbes hampers the pre-infective growth of soil-borne pathogens resulting in lower infection rates (Hoitink and Boehm, 1999). This competition-related mechanism of pathogen control is also known as “general disease suppression” (Hoitink and Boehm, 1999). Disease suppression is closely related to soil fungistasis, the restricted ability of fungal propagules to germinate or grow in most soils (Dobbs and Hinson, 1953). As for general suppression, it has been hypothesized to be caused by microbial withdrawal of nutrients from soil or even from fungal propagules (Lockwood, 1977). However, besides substrate competition, also inhibitory compounds, released by microbes, have been indicated to contribute to fungistasis (Romine and Baker, 1972; De Boer et al., 2003). This implies that not only the carbon-withdrawing activity of the total soil microbial community is involved in fungistasis but also the secondary metabolite production of certain groups within the soil microbial community. Based on this, Garbeva et al. (2011) argued that the composition of soil microbial communities is more important in fungistasis than previously has been appreciated.

Most soil-borne pathogens are poor competitors and have limited saprotrophic capacities in their pre-infective stages. Therefore, they are sensitive to general disease suppression. Among them is the oomycete genus *Pythium*, which includes many plant pathogenic species (Boehm and Hoitink, 1992). They infect roots of seedlings generally resulting in damping-off and, consequently, reduced yield in a broad range of crops (Martin and Loper, 1999). In flower bulb crops, several species of *Pythium* cause severe root rot, leading to considerable losses in bulb yield (van Os et al., 1998). Infection can occur by zoospores and is initiated by a chemotactic response to compounds exuded by roots. Yet, *Pythium* is considered to be a poor competitor for these root exudates and, therefore, natural control of *Pythium* infection is attributed to high competitive pressure exerted by other exudate-consuming soil microbes (Chen et al., 1988; van Os and van Ginkel, 2001). Hence, the current view on the cause of natural buffering of soils against *Pythium* infection is mainly pointing at resource competition rather than at interference competition (involvement of inhibitory secondary metabolites).

Antimicrobial volatile organic compounds (VOCs), emitted by soil microbes, may be an important factor in causing fungistasis facilitated by their ability to diffuse through the porous soil matrix (Wheatley, 2002; Garbeva et al., 2011; Effmert et al., 2012). The potential role of VOCs in suppression of soil-borne plant pathogenic organisms was already reviewed in Stotzky et al. (1976) but regained interest recently (Garbeva et al., 2011; Effmert et al., 2012; Weisskopf, 2013). Production of antifungal volatiles has been shown for a broad range of bacterial phyla: it has been estimated that 30–60% of the soil bacterial species can produce fungistatic volatiles (Wheatley, 2002; Zou et al., 2007). Further support for the role of volatiles in fungistasis came from an extensive inventory by Chuankun et al. (2004), who observed a significant positive correlation between fungistatic activity (inhibition of spore germination) and production of VOCs by 146 soils. The inhibition of pathogen growth by bacterial VOCs has been shown in several studies (McCain, 1966; Alström, 2001; Wheatley, 2002; Kai et al., 2007, 2009; Zou et al., 2007; Effmert et al., 2012) indicating the potential of microbial volatiles in disease reduction. Inhibition of *Pythium* mycelial growth by bacterial volatiles has been shown, albeit under *in vitro* conditions and not in soils (Garbeva et al., 2014a; Hol et al., 2015). Hence, possible involvement of volatiles in natural soil suppression of *Pythium* is unknown.

Agricultural management practices may influence the composition of soil microbial communities and, therefore, also the production of pathogen-suppressing secondary metabolites. Different management practices are in use to reduce pathogen pressure. Anaerobic soil disinfestation (AD) uses crop residues and airtight covering of the soil with plastic foil to stimulate the development of anaerobic microbes producing toxic substances that eliminate harmful nematodes and fungi (Blok et al., 2000). Although AD is used as an environmentally-friendly alternative for chemical disinfestation it is expected to have a tremendous effect on microbial community composition and functioning as aerobic soil microbes face a period of anaerobiosis. Little is known on the possible legacy that AD may have on the composition and functioning of soil microbial communities after the treatment has been finished and cultivation of new crops is started. It has been shown that stress-induced shifts in soil microbial community composition can cause a drastic reduction of fungistasis (De Boer et al., 2003). Hence, there is a potential risk that AD and other disinfestation treatments have similar effects on the pathogen-suppressing activities of soil microbial communities. The current study was aimed to address possible legacy effects of AD of sandy bulb soils on bacterial community composition and soil suppressive characteristics, with special emphasis on the production of pathogen-suppressing volatiles. To this end measurements were done at the start of the flower bulb season (planting of bulbs in autumn) in the year that AD had been applied (3 months after AD) and 1 year later. The oomycete *Pythium intermedium*, a notorious pathogen of flower bulbs, was used to test soil suppressiveness as *Pythium* species are opportunistic pathogens that can rapidly cause problems under conditions where general suppressiveness has been reduced (Postma et al., 2000). Simultaneously the production of *Pythium*-suppressing volatiles by AD-treated and control soils were tested and compared with results of bioassays (root development of Hyacinth bulbs in the presence of *P. intermedium*) to determine the role of volatiles in natural disease suppression. Bacterial community composition was determined using 454 sequencing of 16S rDNA fragments.

The tightly linked series of analyses and experiments lend strong support to the importance of bacterial community composition and–volatile production in natural suppression.

## Materials and methods

### Soil treatments and sampling

Experiments were performed with soil samples from the experimental fields of Applied Plant Research (Wageningen UR) in Lisse, The Netherlands (coordinates: N 52.25. 52; E 4.54. 77). At this location the alluvial sandy soil has a low organic matter content ranging between 1.0 and 1.5%, which is representative of the soil type used for cultivation of flower bulbs along the dunes of the coastal area of the North Sea. In 2010, a field trial was initiated to examine the effect of soil organic matter content and management practices on disease suppression against several soil-borne pathogens. From the current experiment, plots of four soil treatments (Table 1) with four replicates per treatment (60 m^2^ per replicate) were included. In May 2010, organic matter (OM) content was elevated by incorporating a mixture of peat (95%) and cattle manure (5%) (504 tons ha^−1^, 0–30 cm deep), resulting in an increase of the soil OM content from 1.2 to 3.0%. In August 2011, anaerobic soil disinfestation (AD) was applied to a subset of the plots according to the method of Blok et al. (2000) using “Herbie 7025” (van Overbeek et al., 2014), a defined protein-rich vegetal by-product of food processing industry (Thatchtec B.V., Wageningen, The Netherlands). Herbie was applied 24 tons ha^−1^, was incorporated 0–30 cm deep and anaerobic conditions were created by watering followed by airtight covering of the soil with plastic for 6 weeks. Three and fifteen months after removal of the plastic cover (November 2011 and November 2012 respectively), soil samples were taken from each field plot (22 kg per plot, randomly collected from 0 to 20 cm depth) and kept at 4°C until use. In between sampling dates, *Gladiolus* was cultivated on all field plots (April-November 2012).

**Table 1:** **Overview of soil treatments, soil properties, application-, and sampling dates**.

**Code**	**Treatment**	**Organic matter/pH**	**Date of application**	**Plots**	**Sampling dates**
U	Untreated	1.2% pH 7.0	–	4	Nov. 2011, 2012
P	Peat	3.0% pH 7.1	May 2010	4	Nov. 2011, 2012
AD	Disinfested	1.2% pH 6.9	Aug. 2011	4	Nov. 2011, 2012
ADP	Disinfested + Peat	3.0% pH 7.0	Aug. 2011, May 2010	4	Nov. 2011, 2012

### Bioassay for assessment of root rot

From each of the 16 field plots, soil samples were artificially infested with a three-week-old oatmeal culture (1% v/v) of *Pythium intermedium* (isolate P52, Applied Plant Research Flowerbulbs, Nursery Stock and Fruit, Lisse). Non-infested and pasteurized soils (2 h at ≥ 70°C) were used as controls. Soil moisture content was adjusted to 20% (w/w). Five bulbs from *Hyacinthus orientalis* cultivar “Pink Pearl” were planted in pots (3 L) and incubated during 8 weeks at 9°C in the dark in climate cells (Hyacinth bulbs are infected during the belowground root growing phase of the bulb). Pots were sealed with plastic foil to maintain soil moisture but allow oxygen diffusion. Impact of *Pythium* on Hyacinth root development was assessed by measuring root weight and by rating root-rot disease symptoms. At the end of the growing period, bulbs were removed from the soil and roots were washed with tap water. Root-rot ratings of infested treatments were related to the healthy root systems of non-infested control treatments. Roots were visually examined for root rot severity according to van Os et al. (1998) using an arbitrary disease index ranging from 0 to 5, where 0 = no root rot, 1 = 1–20%, 2 = 21–40%, 3 = 41–60%, 4 = 61–80%, and 5 = >80% root rot, i.e., relative loss of healthy root mass induced by infection, compared to the corresponding healthy root systems. Roots were scored for each plant individually and a mean root rot index for each pot was calculated. After the scoring of the disease index, roots were removed from the bulbs and excess water was removed by blotting the roots on filter paper and total fresh root weight per pot (5 bulbs) was determined. Means of four pots per soil treatment were used in statistical analysis. Separate bioassays were performed for both years.

### *In vitro* tests for production of *Pythium*-suppressing volatiles from soil

An experimental set-up was designed to enable exposure of *Pythium intermedium* to volatiles produced by the soils, without direct contact between *Pythium* and soil. Soil samples equal to 20 g dry weight [20% (w/w) soil moisture content] were spread evenly on the bottom of 90 mm Petri dishes and incubated for 1 week at 10°C. A 4 mm layer of water yeast agar (WYA, 20 g agar, 1 g KH_2_O_4_, 0.1 g (NH_4_)_2_SO_4_, 0.1 g yeast extract (Difco) L^−1^ pH 6.5) was poured in lids of Petri dishes. Agar plugs of 6 mm potato dextrose agar (PDA 19,5 g L^−1^ (Oxoid, Basingstoke, UK) with CMN agar 7,5 g L^−1^ (Boom, Meppel, The Netherlands) colonized by *P. intermedium* (incubated 5–10 days at 20°C) were transferred to WYA plates and kept at 10°C. After 48 h, a WYA agar disk (Ø 6 mm) containing *Pythium* mycelium was placed in the center of the lid. The mycelium-containing lid was carefully placed on top of the bottom compartment containing soil and sealed using Parafilm (Figure S1). Plates were incubated for 10 days at 10°C. Petri dishes without soil and with gamma-radiated soil (untreated 2012) (>25 kGray, Isotron, Ede, the Netherlands) were used as controls for conditions without microbially produced volatiles. Before the start of the experiment the gamma-radiated soil was left for 4 days in a sterile flow cabinet to remove all residual volatiles. Mycelial biomass determination was done according to the method of Garbeva et al. (2014b) with some modifications. Briefly, *Pythium* mycelia were harvested by melting the colonized agar from the lids of the Petri-dishes in a beaker glass with water in a microwave oven (c. 100°C), followed by sieving with a tea strainer and three washing steps with water (c. 90°C) in order to remove agar residues. For measurements of dry biomass weight, mycelia were frozen at −20°C and freeze-dried during 24 h. Pictures of *Pythium* hyphae were taken before harvest 1 cm from the edge of the plate with a stereo microscope (Olympus, SZX12, Tokyo, Japan) connected to a AxioCam MRC5 camera (Zeiss, Jena, Germany) under a 90x magnification.

### Trapping and GC/MS analysis of microbial volatiles

For collection and analysis of released volatiles from soil the method of Garbeva et al. (2014b) was used with some modifications. Soil from two plots per treatment was randomly selected for GC/MS analysis. Briefly, soil samples were plated in special designed glass petri dishes, with an exit to which a steel trap could be connected with as trapping material 150 mg Tenax TA and 150 mg Carbopack B (Markes International Ltd., Llantrisant, UK) which could fix VOCs released from the soil. VOCs were collected after 168 hours of incubation at 10°C. Then, traps were removed, sealed and stored at 4°C until further analysis. Volatiles were desorbed from the traps using an automated thermodesorption unit (model Unity, Markes International Ltd., Llantrisant, UK) at 200°C for 12 min (He flow 30 ml/min). Each trap was heated for 3 min up to 270°C to introduce the volatiles into the GC/MS (model Trace, ThermoFinnigan, Austin, TX, USA). Split ratio was set to 1:4, and the used column was a 30 × 0.32 mm ID RTX-5 Silms, film thickness 0.33 μm (Restek, Bellefonte, PA, USA). The used temperature program was: from 40°C to 95°C at 3°C min^−1^, then to 165°C at 2°C min^−1^, and to 250°C at 15°C min^−1^. The VOCs were detected by the MS operating at 70 eV in EI mode. Mass spectra were acquired in full scan mode (33–300 AMU, 0.4 scans s^−1^). Compounds were identified by their mass spectra using deconvolution software (AMDIS) in combination with NIST 2008 (National Institute of Standards and Technology, USA,), Wiley 7th edition spectral libraries and by their linear retention indices (lri). The lri values were compared with those found in the NIST and the NIOO lri database. Candidate compounds possibly related to volatile inhibition of *Pythium* growth were identified by screening for volatiles that were absent in disinfested soils (AD and ADP) in 2011 and present 1 year later (2012) and in non-disinfested soils (U and P) 2011 and 2012.

### Pyrosequencing of soil bacterial communities

DNA was extracted directly after sampling from three randomly selected plots per treatment using Mobio 96 well Powersoil® extraction kit according to the manual. Amplicons for barcoded 16S pyrosequencing were generated using PCR reactions (5 min 95°C followed by 25 cycles 95°C 30 s, 53°C 60 s, 72°C 60 s + 1 s per cycle finishing with 10 min 72°C and 10°C soak) performed in triplicate for each sample using the primerset 515F (5'-GTGCCAGCMGCCGCGGTAA-3') and 806R (5'-GGACTACVSGGGTATCTAAT-3') (Caporaso et al., 2011). The 515F primer included the Roche 454-pyrosequencing adapter and a GT linker, while 806R included the Roche 454- sequencing adapter, a 12-bp barcode (unique to each sample), and a GG linker. PCR products were cleaned (Qiagen Pcr purification kit) pooled and were sequenced (Macrogen Inc. Company, South Korea) on a Roche 454 automated sequencer and GS FLX system using titanium chemistry (454 Life Sciences, Branford, CT, USA). The obtained 454 sequences were filtered and analyzed using QIIME (Caporaso et al., 2010) in the Galaxy interface. Sequences were denoised (DENOISER, Reeder and Knight, 2010) and chimeras were removed using UCHIME (Edgar et al., 2011) followed by trimming of low quality reads (<200 bp, quality score 20). The remaining high quality sequences were clustered into operational taxonomic units (OTU's) using UCLUST (Edgar, 2010) with a minimal sequence identity cut off of 97% using the most abundant unique sequence as cluster representative. Sequences were deposited in the European Nucleotide Archive under accession number PRJEB6155 (http://www.ebi.ac.uk/ena/data/view/PRJEB6155).

### Data analysis

In the bioassay, mean disease indices per pot were converted to percentages. The assumption of normality was tested with Shapiro–Wilk statistics and Levene's test was used to confirm homogeneity of variances. An analysis of variances, a Three-Way ANOVA, was performed to test the effects of soil treatment, *Pythium* addition, organic matter level and their interactions on root weight or percentage root rot.

To test the effects of VOC produced in soil on *Pythium* biomass the average hyphal weight per Petri dish per soil treatment was determined in 2011 (*n* = 16) and 2012 (*n* = 8). Data were calculated as percentage of the growth of the control. Normality was tested with Shapiro-Wilk test and homogeneity of variances was assessed with Levene's test. A Two-Way ANOVA was performed to determine differences between soil treatments.

A Three-Way ANOVA was performed on the data from the pyrosequencing analysis in order to test the effects of peat addition, soil treatment and sampling year and their interactions on number of reads. Pyrosequencing data were rarefied to the lowest number of obtained reads, 2047 reads per sample. Per phylum all soil treatments in the two seasons were tested for a change in relative abundance, based on the number of reads per phylum. The average number of reads per phylum per soil treatment was calculated and tested with One-Way ANOVA. The average number of OTUs per treatment was used to express OTU richness. Although the OTU richness data did not meet the assumption of normality in the analysis these data were also analyzed with a One-Way ANOVA to determine differences between treatments. Statistical analyses were done in R3.0.2 and PAST (Hammer et al., 2001).

## Results

### Impact of *Pythium* on hyacinth root rot (bioassays)

**Figure 1:**
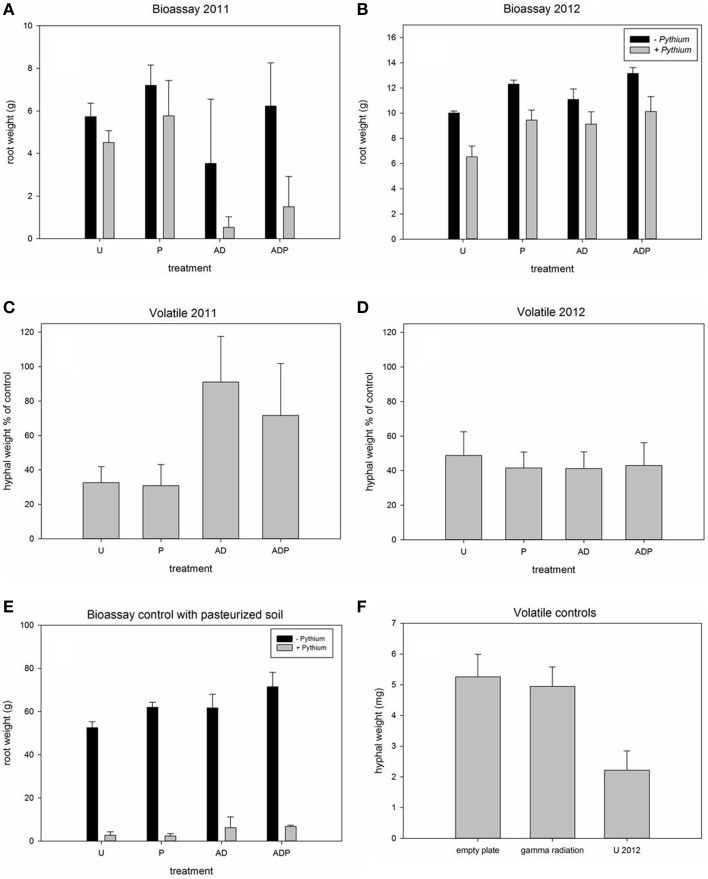
**Root biomass of Hyacinth bulbs in soils with and without addition of *Pythium intermedium* and production of hyphal biomass by *P. intermedium* during exposure to soil volatiles**. **(A,B)** Average weight of roots extending from Hyacinth bulbs grown in differently managed soils (*U*, untreated; *P*, peat addition; *AD*, anaerobic disinfestation) with and without *Pythium* addition. **(C,D)** Average hyphal weight of *P*. *intermedium* hyphae that had been exposed to volatiles produced by differently managed soils. *Pythium* biomass is presented as percentage of the empty plate control. **(E,F)**, control experiments: **(E)**, Bulb root weight in pasteurized soils with and without addition of P. intermedium; **(F)**, Average hyphal weight of empty plates, gamma irradiated soil and the untreated soil in 2012. Significant results of main treatment effects and interactions are presented in Table 2, marked in bold. Error bars represent standard deviation.

**Figure 2:**
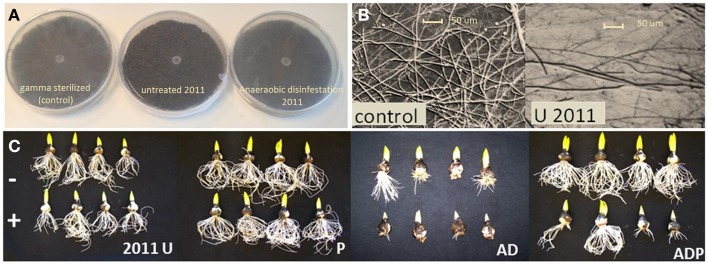
***Pythium* volatile exposure assays and Hyacinth bioassays. (A)** Differences in *Pythium* hyphal density upon volatile exposure sterilized soil (control) and untreated and disinfested soil in 2011. **(B)** Detailed pictures taken from agar plates after volatile exposure. **(C)** Results of Hyacinth bioassays in soil from differently managed fields without (−) and with (+) *Pythium intermedium* addition. U, untreated; P, peat addition; AD, anaerobic disinfestation.

Management practices strongly affected root biomass and root rot severity. Addition of *Pythium* to the soils showed an overall effect of the pathogen: the root weight was significantly reduced in all soils in the consecutive years. However, the magnitude of the effect of *Pythium* on root biomass reduction was different depending on the management regime the soil had received (Figures 1A,B, 2C). *Pythium*-induced root biomass reduction was most strong in recently (2011) anaerobic disinfested (AD) soils. This was also indicated by the significant interaction between soil disinfestation and *Pythium* addition in 2011 (Table 2). In contrast to 2011, the effect of *Pythium* addition to the soil in 2012 was independent of the former AD treatment and did not show differences between the differently managed soils (Figure 1B). In all pasteurized soil samples, inoculation with *Pythium* resulted in a severe loss of root biomass, average root weight was reduced by >60% (Figure 1E).

**Table 2:** **Analysis of variance for root biomass and disease indexes of hyacinth bulbs in the soil bioassays and hyphal biomass in the volatile exposure assays**.

	**Df**	**F 2011**	**p 2011**	**Df**	**F 2012**	**p 2012**
**ROOT WEIGHT**						
Anaerobic soil disinfestation	1	26.24	**3.1E–05**	1	22.77	**7.4E–05**
Peat addition	1	8.23	**8.5E–03**	1	57.63	**7.9E–08**
*Pythium* addition	1	21.64	**1.0E–04**	1	107.4	**2.4E–10**
Disinfestation: peat amendment	1	0.18	0.68	1	3.92	0.06
Disinfestation: *Pythium* addition	1	5.19	**0.03**	1	1.60	0.22
Peat amendment: *Pythium* addition	1	0.76	0.39	1	0.18	0.68
Disinfestation: peat: *Pythium* addition	1	0.45	0.51	1	2.42	0.13
**VOLATILE ASSAY**						
Anaerobic soil disinfestation	1	85	**4.0E–13**	1	0.58	0.45
Peat addition	1	4	0.05	1	0.43	0.52
Disinfestation: peat amendment	1	3	0.11	1	1.17	0.29
**DISEASE INDEX**						
Anaerobic soil disinfestation	1	54.62	**1.3E–07**	1	2.74	0.11
Peat addition	1	4.52	**0.04**	1	17.38	**3.4E–04**
*Pythium* addition	1	5.81	**0.02**	1	125.09	**5.3E–11**
Disinfestation: peat amendment	1	5.07	**0.03**	1	1.27	0.27
Disinfestation: *Pythium* addition	1	5.51	**0.03**	1	1.93	0.18
Peat amendment: *Pythium* addition	1	0.20	0.66	1	6.00	**0.02**
Disinfestation: peat: *Pythium* addition	1	0.91	0.35	1	0.22	0.65

*Bold numbers indicate significant main effects and significant interactions. Df, degrees of freedom; F, F-value; p, p-value. This table supplies the results of statistical analysis of the data shown in **Figure 1***.

Root biomass in the bioassays was not only affected by *Pythium* but also by the different management practices as became apparent from the control bioassays, i.e., the pots without *Pythium* addition. Both peat amendment and soil disinfestation significantly affected root weight in both years of sampling (see Figures 1A,B, 2C; Table 2). Addition of organic matter significantly increased the root weight in both years; the root biomass was significantly higher in peat-amended soils (2011, 21% for P and 43% for ADP; 2012 19% for P and 16% for ADP) than in the comparable soils without peat addition. Soils that had received a recent AD treatment had a significantly reduced root weight, 39% (AD) and 14% (ADP), as compared to the untreated (U) and peat-amended soil (P) respectively. One year later this effect was reversed. In 2012, plants in formerly anaerobic disinfested soils had a higher root biomass, 10% and 7% more, compared to the non-disinfested soils with the same organic matter level. Similar to the effects of *Pythium* on root weight, anaerobic disinfestation enhanced the effects of *Pythium* on root rot symptoms in 2011 (Figure S2A; Table 2). This interaction was no longer apparent in 2012 (Figure S2B; Table 2). Even without addition of *Pythium*, an increase of root rot symptoms was found for recently disinfested soils (Figure S2A; Table 2). Peat addition reduced the severity of root-rot symptoms significantly (Figure S2B, Table 2). The infective ability of the applied *Pythium* inoculum was confirmed, as a strong increase of root rot symptoms was seen in pasteurized soil (Figure 1E).

### Emission of *Pythium*-inhibiting volatiles by soils and soil microbes

Exposure of *Pythium* to volatiles released from the soils resulted in a strong reduction of *Pythium* biomass production (Figures 1C,D, 2A,B). There were, however, differences between treatments and sampling years. Compared to the empty plate control, both the untreated soil and soil with peat amendment gave a 3-fold reduction in mycelial biomass in 2011 (*p* < 0.0001) (Figures 1C,D; Table 2). In contrast, exposure of *Pythium* to volatiles released from the anaerobic disinfested soils did not (AD) or only slightly (ADP) result in reduction of *Pythium* biomass or hyphal density (Figures 1C,D, 2A,B; Table 2). In 2012, this lack of volatile suppression in disinfested soils was no longer apparent as volatiles from all soils significantly reduced *Pythium* growth by at least 50% compared to soils (Figures 1D, 2A,B). The impact of soil-derived volatiles on *Pythium* growth was not significantly affected by peat addition. Volatile-suppression of *Pythium* growth was not seen when exposed to gamma-radiated soils (Figure 1F), indicating that no growth-reducing volatiles were produced in soil without microflora.

### Trapping and GC/MS analysis of bacterial volatiles

GC/MS analysis identified >700 different volatile compounds that were released from the soil of which 15 compounds were found to be absent in the anaerobic disinfested soil in 2011 (Table 3), mostly ketones. Some of these compounds, namely 2-octanone, 2-undecanone and 2-nonanone, are known to be inhibitors of eukaryotic pathogenic soil organisms (Table 3). Besides ketones the 15 potential suppressive compounds included glycol ethers, alkanes, a fatty acid and two yet unidentified compounds with retention indices of 1692 and 1743. One year later, the 15 volatile compounds were again released by the previously disinfested soils.

**Table 3:** **Volatile organic compounds of which the production appeared to be negatively affected by the anaerobic disinfestation treatment in 2011**.

**2011**	**2012**							
	**U**	**P**	**AD**	**ADP**	**U**	**P**	**AD**	**ADP**
**2-octanone^a^**	**−**	**+**	**−**	**−**	**+**	**+**	**+**	**+**
**2-nonanone^b^,^c^**	**+**	**+**	**−**	**−**	**+**	**+**	**+**	**+**
**2-undecanone^c^,^d^**	**+**	**−**	**−**	**−**	**−**	**+**	**+**	**+**
2-hexanone	−	+	−	−	+	−	+	+
2-tetradecanone	+	−	−	−	−	+	−	+
2,5-hexanedione	−	+	−	−	+	−	+	−
1-octen-3-one	+	+	−	−	+	+	−	+
1-butoxy-2-propanol	+	+	−	−	+	+	+	+
2-butoxyethanol	−	+	−	−	+	+	+	+
hexadecane	+	+	−	−	+	+	+	+
1-nonanol	+	+	−	−	+	+	+	−
nonylcyclohexane	−	+	−	−	+	+	+	+
heptanoic acid	−	+	−	−	+	+	+	+
unknown 1692a^*^	+	+	−	−	+	+	+	+
unknown 1743a^**^	+	+	−	−	+	+	+	+

*Bold compounds have been previously identified as potential fungus suppressing compounds. Chemical names are according IUPAC*.

* *Unknown 1692a Lri: 1692; EI: 88 (100), 121 (75), 174 (10)*.

** *Unknown 1743a Lri: 1743; EI: 104(100), 78(11), 208(3)*.

a *Zou et al. (2007); inhibiting concentration not given*.

b *Chen et al. (2008); inhibiting concentrations not given*.

c *Weisskopf (2013), inhibiting concentrations not given*.

d *Fernando et al. (2005), inhibiting concentration not given*.

### Pyrosequencing of soil bacterial communities

**Figure 3:**
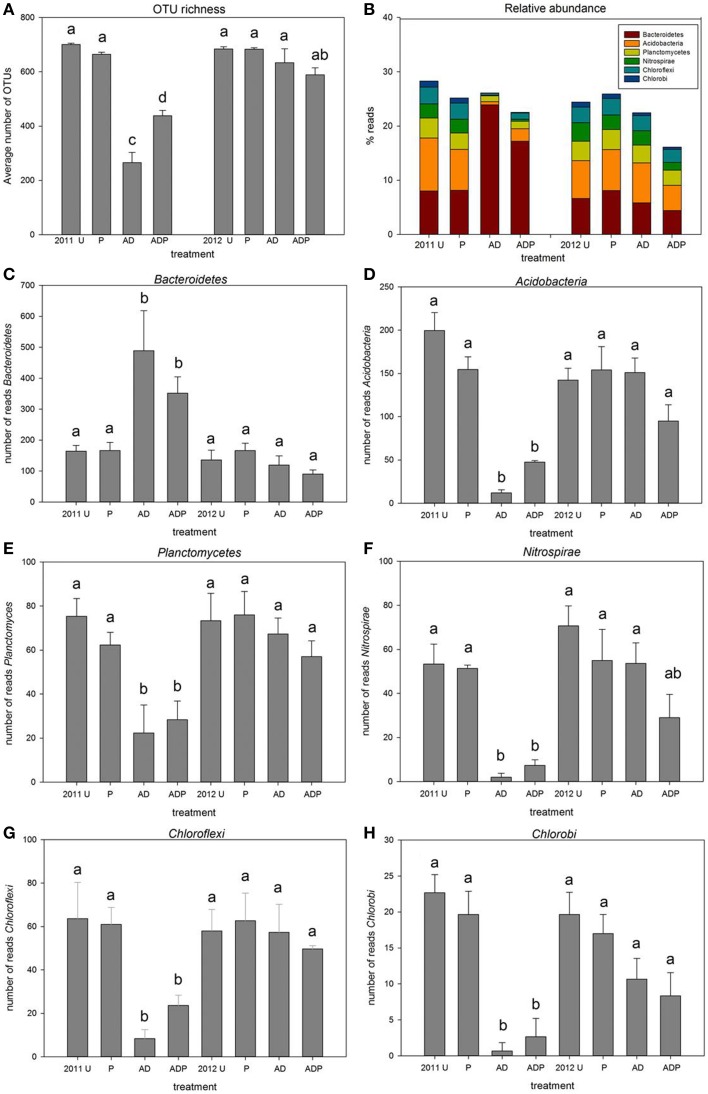
**OTU richness and average relative abundance of selected bacterial phyla. (A)** average number of OTUs in differently managed soil (*n* = 3, error bars represent stdev, *U*, untreated; *P*, peat addition; *AD*, disinfestation), **(B)** relative abundance **(C–H)**, average relative abundance of phyla that differ significantly between the disinfested soils in 2011 (AD and ADP) and all other treatments in 2011 and 2012.

Four hundred fifty four Pyrosequencing identified over 3000 different OTUs from 31 bacterial and 2 archaeal phyla (Figure 3A). There was no soil treatment effect on the number of reads obtained per sample, average numbers of reads were not different between soil treatments or sampling years (Figure 3B). In 2011, anaerobic soil disinfestation had resulted in reduction of OTU richness. At higher organic matter level the reduction was significantly less (Figure 3A). In 2012 no differences in OTU richness were seen between soil treatments. Most abundant (36–63%) in all samples were OTUs assigned to *Proteobacteria*. Relative abundances of classes within the *Proteobacteria*, i.e., *Alpha- Beta- Gamma-* and *Deltaproteobacteria*, did not change significantly between different soil treatments (Figure S3). In 2011, six phyla showed significant differences between plots with and without anaerobic soil disinfestation (Figures 3C–H). At the start of the bulb planting season for spring flowering bulbs anaerobic soil disinfestation had still clear effects on the bacterial community composition. Relative abundances of OTUs assigned to *Acidobacteria, Chloroflexi, Nitrospirae, Chlorobi*, and *Planctomycetes* were significantly lower in the disinfested soils, whereas relative abundance of OTUs assigned to *Bacteroidetes* was higher compared to untreated soils. In 2012, 15 months after the disinfestation treatment, the relative abundance of these phyla was restored to the same levels as occurred in untreated soils for *Acidobacteria, Chlorobi* and *Planctomycetes* (*p* < 0.05), with the same tendency for *Chloroflexi* and *Nitrospirae* (*p* < 0.1) (Figure 3, Table S1).

## Discussion

Volatile organic compounds form an important part of the underground chemical communication network between plants, fungi and bacteria (Ryu et al., 2003; Vespermann et al., 2007; Insam and Seewald, 2010; Effmert et al., 2012; Bitas et al., 2013; Fiers et al., 2013). They can have different roles in the soil including plant growth promotion and signaling (Vespermann et al., 2007). There are also indications that VOCs produced by soil micro-organisms can have an important contribution to the restriction of growth and germination of pathogenic fungi (fungistasis) that occurs in most soils (Garbeva et al., 2011). However, despite the ability of several soil microbial VOCs to reduce pathogenic growth; little attention has been paid to the role of these VOCs in suppression of plant diseases caused by soil-borne pathogens.

Our results provide an indication about the involvement of VOCs in natural disease suppression of a soil-borne pathogen. Our study revealed interesting co-incidences of severe *Pythium*-induced root weight loss, absence of production of *Pythium* suppressing soil volatiles and shifts in bacterial community composition shortly after an anaerobic disinfestation treatment. One year later these effects of the disinfestation treatment had largely disappeared. The coinciding dynamics of root biomass and production of suppressing volatiles suggests that microbial volatiles can have an important contribution in the natural control of *Pythium intermedium*. Furthermore, our results point at the importance of microbial community composition as disinfestation-induced shifts in community composition which coincided with the loss in suppressiveness by volatiles.

Induced changes in microbial community composition can yield important information on the functioning of the original soil microbial communities (Griffiths and Philippot, 2013). Management practices can alter the abundance of microbial groups that are thought to be involved in disease suppression (Garbeva et al., 2004; Mazzola, 2004; Chaparro et al., 2012; Sipilä et al., 2012). In the current investigation we observed that anaerobic soil disinfestation had a dramatic effect on soil microbial diversity and community composition. This is not surprising as the microbes in the well-drained sandy soils were confronted with a long period of oxygen-depletion resulting in a shift from aerobic metabolism to predominant anaerobic metabolism. The impact of this period of anaerobiosis was still clearly visible in the bacterial community composition at the time that flower bulbs are usually planted i.e., 3 months after the soil disinfestation treatment had been ended (removal of cover plastic). Most striking was the high abundance of *Bactereroidetes*, a phylum that has been shown to strongly respond to fluctuating redox conditions (DeAngelis et al., 2010).

In contrast, the phyla *Acidobacteria, Planctomycetes, Nitrospirae, Chloroflexi*, and *Chlorobi* decreased significantly after disinfestation. So far, these groups have rarely been studied in the context of disease suppression, due to limitations to obtain cultivable representatives. Strongest reduction in relative abundance of OTUs after soil disinfestation was seen for *Acidobacteria*. A recent study showed that *Acidobacteria* were one of the groups that were most sensitive to a strongly disturbing soil treatment (fumigation) (Domínguez-Mendoza et al., 2014). Earlier studies have shown that changes in land use, fertilization and management caused shifts in relative abundance of *Acidobacteria* and its different subgroups (Jones et al., 2009; Barnard et al., 2013; Navarrete et al., 2013). However, the impact of such shifts in abundance of *Acidobacteria* on disease suppressiveness has not been examined. Yet, next to our results a study by Hunter et al. (2006) provides indications for a possible role of *Acidobacteria* in disease suppressiveness. In that study it was observed that *Acidobacteria* were present in peat suppressive to *P. sylvaticum* whereas they were absent in peats that were conducive for *P. sylvaticum* damping-off. No other documentation is available on antagonistic roles of *Acidobacteria*, nor on production of antimicrobial VOCs. Our data suggest a potential role in the production of suppressing volatiles by *Acidobacteria*. However, the actual role of *Acidobacteria* in volatile production and disease suppression, as well as that of the other phyla that showed similar dynamics upon disinfestations remains to be established.

Remarkably, the main classes of *Proteobacteria*, which contain many known potential biocontrol bacteria (Weller et al., 2002; Haas and Defago, 2005) did not change significantly as a result of the disinfestation treatment (Figure S3). However, this does not necessarily imply that *Proteobacteria* did not contribute to *Pythium* disease suppression. Mendes et al. (2011) compared a soil suppressive against the plant pathogen *Rhizoctonia solani* with another, similar soil that was conducive for disease caused by this fungus and found similar abundances for all classes of *Proteobacteria* in both soils. However, at species level, e.g., within the genus *Pseudomonas*, differences were observed with higher abundance of antibiotic-producing species in the suppressive soil.

Comparison of the volatiles produced by differently treated soils revealed potential *Pythium*-inhibiting compounds, mostly methyl ketones. These VOCs were present in the untreated and peat-amended soils that exhibited a high level of natural suppression against *Pythium*, but absent in the recently disinfested soils that were susceptible to infection of bulbs by *Pythium*. Among these VOCs, there were compounds like 2-octanone, 2-nonanone and 2-undecanone that were previously found to be suppressive against soil fungi and nematodes (Chen et al., 1988; Alström, 2001; Wheatley, 2002; Gu et al., 2007; Kai et al., 2007; Effmert et al., 2012). Thus these findings support the potential suppressive role of the VOCs identified in this study. However, the antimicrobial role of the other potential suppressive compounds identified in this study remains to be assessed. Besides the compounds highlighted in this study (Table 3) it is possible as well that a mixture of different compounds (Tunc et al., 2007; Veras et al., 2012), compounds that were not detected with the chosen method and/or concentration dependent effects (Wheatley, 2002) are responsible for the suppression against *Pythium*. The biological and ecological relevance of concentration effects and volatile mixture compositions remains to be studied for the natural soil habitat.

Anaerobic soil disinfestation is applied to kill a broad range of pathogens (Blok et al., 2000). The demonstrated reduction of disease suppression shows that such a drastic treatment of the soil has the risk of a (partial) elimination of the natural suppressive microflora. After one growing season, 15 months after application, disease suppression against *Pythium* was restored to the level of the non-disinfested plots. Similar loss of suppressing activity of the indigenous microflora has been found with pathogen-eliminating measures like flooding (8 weeks) and chemical soil disinfestation with cis-dichloropropene or methylisothiocyanate (van Os et al., 1999). Postma et al. (2000) observed enhanced *Pythium* outbreaks in cucumber grown on rockwool after sterilization of the rockwool and recolonisation by a microbial community which lacked the suppressive properties of the original community. Hence, (temporal) changes in the suppressive community, by reducing the competition pressure or elimination of useful microbes, can enhance disease outbreaks of opportunistic pathogens such as *Pythium* (van Os et al., 1999; van Os and van Ginkel, 2001). Our study included two consecutive years of bulb planting to determine the longer term effect of the management treatments on *Pythium* suppression. Fifteen months after the disinfestation treatment, the bacterial community composition resembled the composition of the non-disinfested soils. This is in agreement with the results of Mowlick et al. (2013) who found a restoration of the original microbial community composition in the biological disinfestation treatment after plant growth. Anaerobic disinfestation had an impact on the taxonomic composition of the soil microbial community but also on an important function, namely disease suppression. In our study, after the cultivation of the summer crop *Gladiolus* the suppression of *Pythium* in both volatile assay and bioassay also returned to the level of that in non-disinfested soils. This recovery of the natural suppression against *Pythium* indicates resilience of the soil to re-establish this essential ecosystem function after a strong disturbance (Griffiths and Philippot, 2013).

In the year of application (2011), the anaerobic soil disinfestation had a negative effect on Hyacinth root development even without addition of *Pythium*. This may be the result of phytotoxic effects of compounds that have been produced during anaerobic decomposition and were still present. It is known that decomposition of crop residues during the period of oxygen depletion can produce phytotoxic compounds (Bonanomi et al., 2007b). Since this reduced root growth was only significant in the disinfested soil without peat addition, the increased organic matter levels in peat-amended soils may have absorbed possible phytotoxic compounds.

Organic amendments do influence soil physical-chemical properties as well as soil microbial activity and composition (Hoper and Alabouvette, 1996; Bonanomi et al., 2007a, 2010). Therefore, we expected to find an effect of peat addition on volatile suppression. The addition of peat increased root weight and reduced root rot symptoms in 2012 as compared to the untreated soil. However, the volatile suppression of peat-amended soils was not different from that of the unamended soils. This is in line with the microbial community composition which was not strongly affected by peat amendment but does imply that other mechanisms of disease suppression, besides volatiles, contribute to disease suppression after peat addition. It is clear that organic amendments and disease control measures can have long-term effects on both the soil microflora and on disease suppression, although the effects of the amendments might depend on the nature and maturity of the organic additions (Hoitink and Boehm, 1999; Termorshuizen et al., 2006). In order to get more understanding of time-related changes it is necessary to monitor these soil characteristics during longer periods of time. Since flower bulb production, and more general arable agriculture, are not only seriously threatened by *Pythium*, but also by several other soil-borne fungi and nematodes, is would be recommendable to extend these studies to include also other pathogens like *Rhizoctonia solani, Pratylenchus penetrans* or *Meloidogyne hapla*. In conclusion, our study indicates that the production of suppressing volatiles by soil microbes may be an important factor in the natural suppression of root-infection by *Pythium*. More general, this indicates that microbial volatiles may be an essential part of the natural buffering of soils against soil-borne diseases, the so-called general disease suppression. This would open new perspectives and insights for the control of soil-borne pathogens. Volatile-inhibition tests as well as the presence of certain VOCs and microbial groups could be an indicator of the susceptibility of a given soil to soil-borne pathogens. Obviously, more research is needed to find support for this. In depth studies are needed to further assess the role of volatiles in disease suppression and should also consider the dynamics of production of VOCs in soils, as well as the conditions that affect the sensitivity of the pathogens to VOCs.

### Conflict of interest statement

The authors declare that the research was conducted in the absence of any commercial or financial relationships that could be construed as a potential conflict of interest.
