# Supplementary material for: Legacy effects of anaerobic soil disinfestation on soil bacterial community composition and production of pathogen-suppressing volatiles
Source: Front Microbiol. 2015 Jul 10;6:701. doi: 10.3389/fmicb.2015.00701 (PMC4498103; doi:10.3389/fmicb.2015.00701)
Supplement: Supplementary file 1 [file Table1.DOCX]

**Supplementary material:**

**Table S1** *p* values of the analysis of variance for different bacterial phyla that significantly differ between the management treatments and sampling years (U= untreated, P = peat addition, AD = disinfestation)

**Figure legends**

**Figure S1** Illustration of the petri dish set-up used to determine the effect of soil-derived volatiles on *Pythium* biomass. Bottom petri dish compartments contain soil. Lid compartments contain water yeast agar with in the middle a *Pythium* plug as inoculum.

**Figure S2** Percentage root rot of Hyacinth roots in soils with and without addition of *Pythium* *intermedium.* **a** and **b**, Average percentage of roots extending from Hyacinth with root rot symptoms with and without *Pythium* addition in differently managed soils (U= untreated, P = peat addition, AD = disinfestation) Significant changes and interactions are presented in Table Error bars represent standard deviation.

**Figure S3** Average relative abundance of *Proteobacterial* OTUs (U= untreated, P = peat addition, AD = disinfestation).
