# Supplementary figures and images for: Legacy effects of anaerobic soil disinfestation on soil bacterial community composition and production of pathogen-suppressing volatiles
Source: Front Microbiol. 2015 Jul 10;6:701. doi: 10.3389/fmicb.2015.00701 (PMC4498103; doi:10.3389/fmicb.2015.00701)

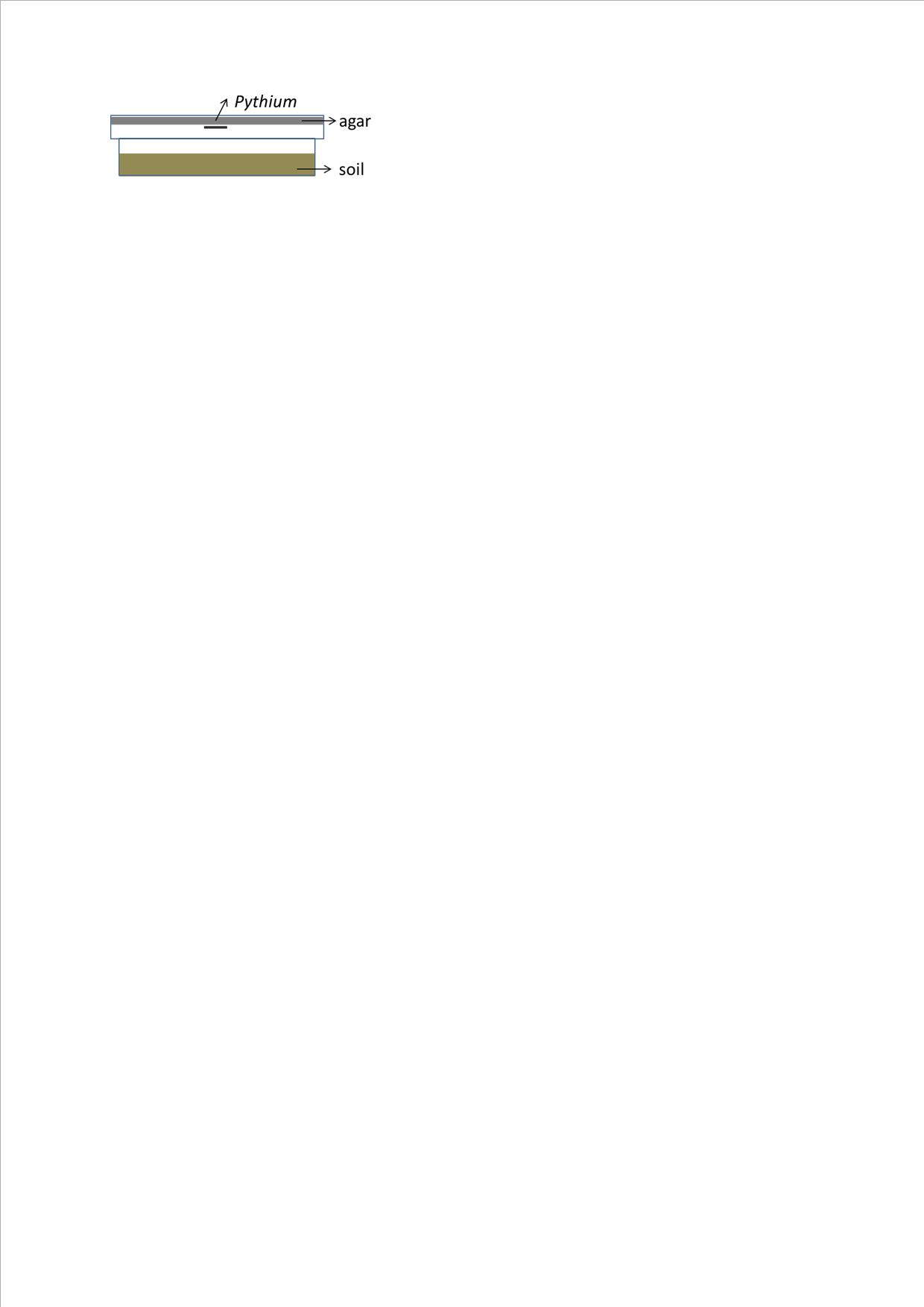

Supplement: Supplementary file 2 [file Image1.JPEG]

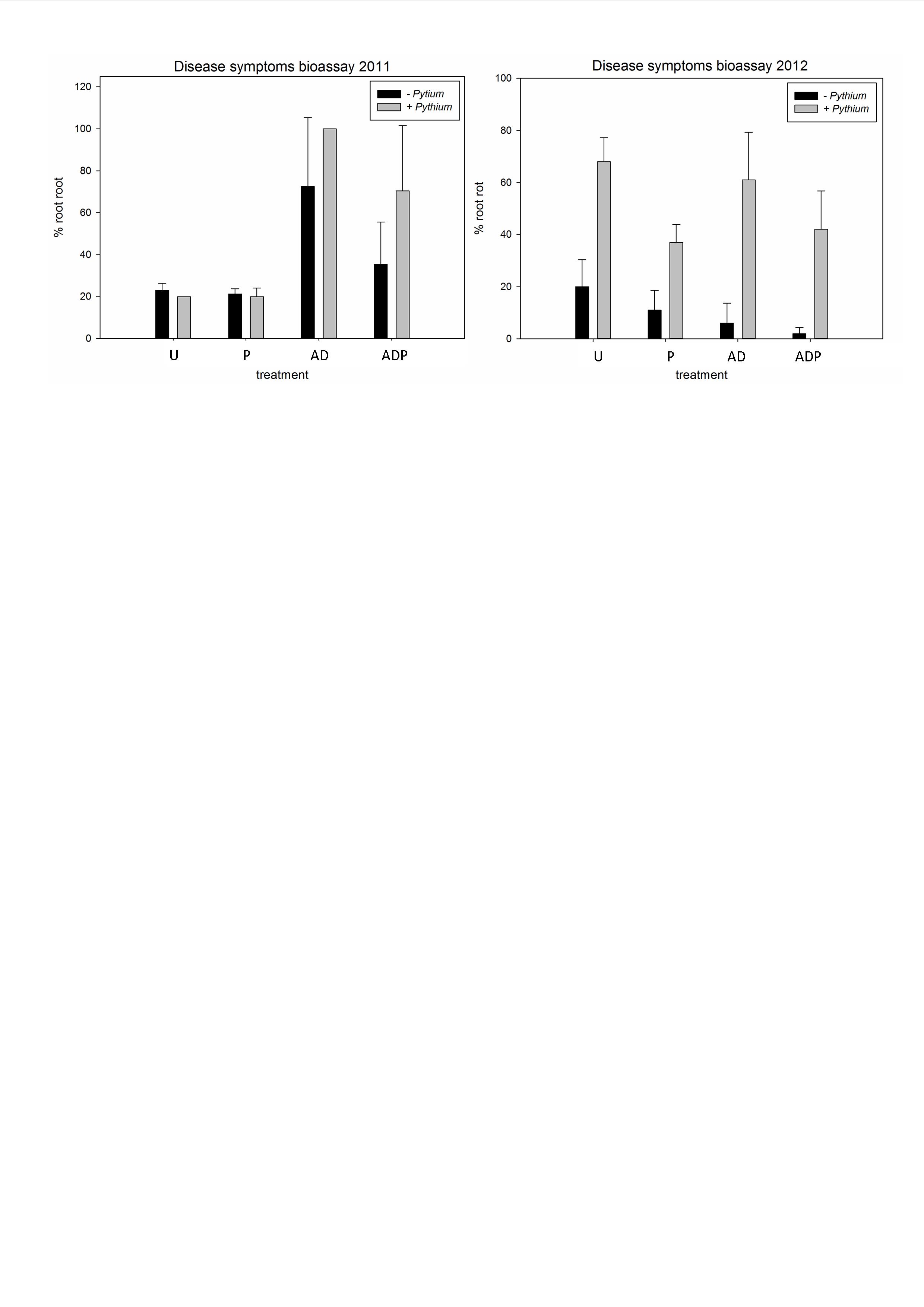

Supplement: Supplementary file 3 [file Image2.JPEG]

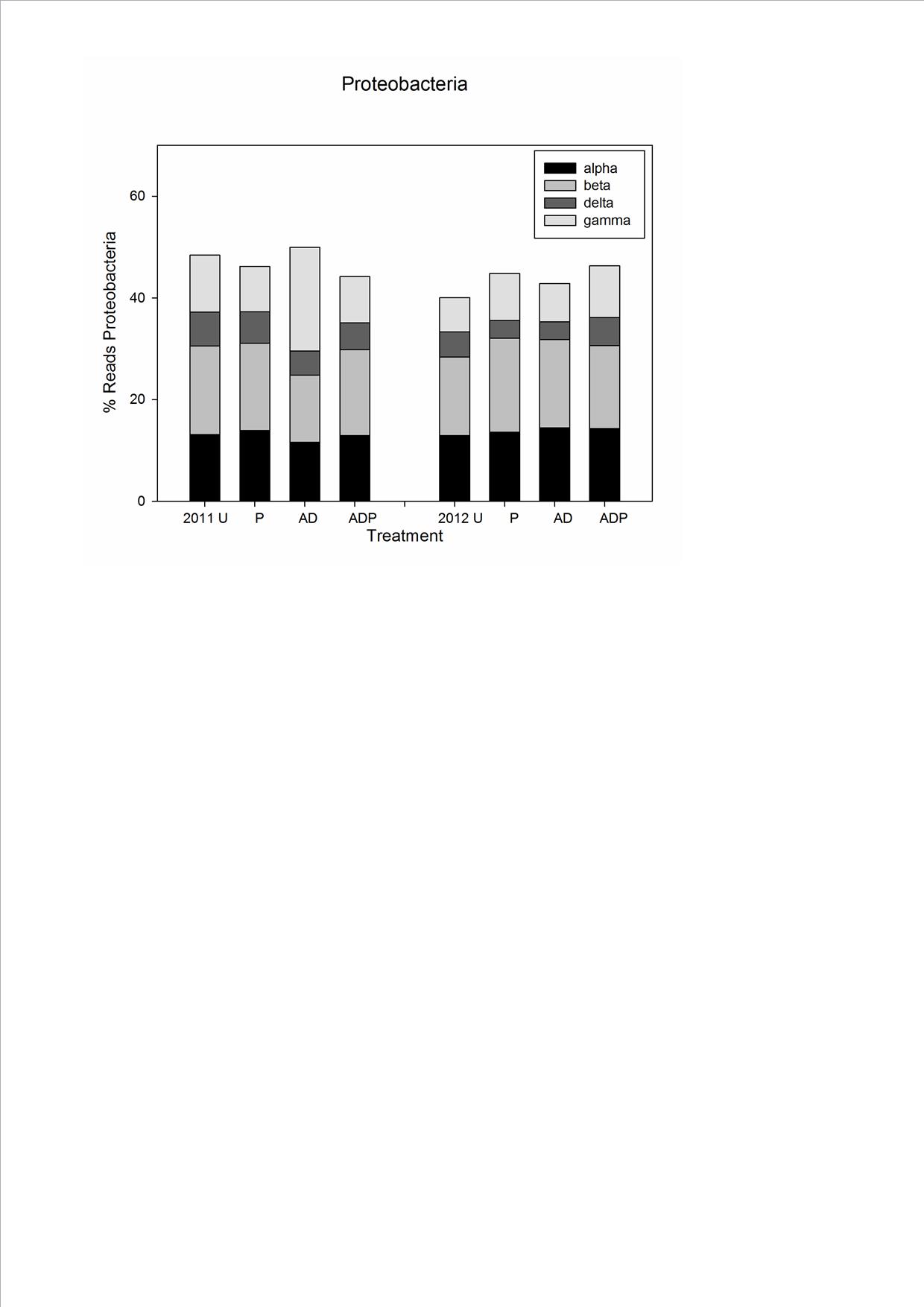

Supplement: Supplementary file 4 [file Image3.JPEG]
